# Intrauterine inoculation of pseudorabies virus impairs mouse embryo implantation via inducing inflammation and apoptosis in endometrium

**DOI:** 10.3389/fvets.2024.1475400

**Published:** 2024-10-31

**Authors:** Zhiqiang Chen, Yuting Wen, Lizhou Meng, Suyao Li, Wenpeng Min, Anwen Yuan, Wen-Lin Chang, Qing Yang

**Affiliations:** ^1^Department of Clinical Veterinary Medicine, College of Veterinary Medicine, Hunan Agricultural University, Changsha, China; ^2^College of Medical Technology and Nursing, Hunan Institute of Traffic Engineering, Hengyang, China; ^3^Department of Obstetrics, The People's Hospital of Longhua, Shenzhen, China

**Keywords:** pseudorabies virus, intrauterine inoculation, embryo implantation, endometrium, inflammation, apoptosis

## Abstract

Pseudorabies virus (PRV) is the pathogenic agent of pseudorabies, causing serious reproductive failure in swine. However, it is still unknown whether PRV uterine inoculation impairs blastocyst implantation. In the present study, a PRV infection mouse model was developed. Pregnant mice were inoculated with either 10^4^ or 10^5^ 50% tissue culture infectious dose (TCID_50_) units of PRV on gestation day 2 (GD2). Viral DNA was detected in tissues by PCR and/or *in situ* hybridization. Histopathological change and expression of proinflammatory cytokines in uterus were analyzed by H.E. staining and qPCR, respectively. Apoptosis was also investigated by TUNEL assay, and the expression of apoptosis-related proteins including Bax and Bcl-2 was detected by Western blot. The results showed that intrauterine exposure of PRV on GD2 reduced the number of embryo implantation site. Abundant viral DNA was detected in spinal marrow and brain, and small amounts of PRV genomes were detected in embryo implantation site, ovary as well as thymus. Considerable inflammatory cells infiltrating in the endometrium, with high levels of pro-inflammatory cytokines of interleukin (IL)-6, IL-1β and tumor necrosis factor-*α* mRNA after infection. In addition, PRV infection promoted apoptosis in stroma and endothelium of the mouse endometrium. Collectively, intrauterine inoculation of PRV during early pregnancy causes cytokine release syndrome and apoptosis in endometrium, which impairs mouse embryo implantation.

## 1 Introduction

Pseudorabies virus (PRV) is a member of alpha-herpes virus family. PRV infection causes estrus return, abortion, and birth of weak or dead piglets in sows (1, 2). Intact zonae pellucida plays a major role in protecting the preimplantation embryos against virus infection. Porcine embryonic cells up to 16-cell stage were resistant to PRV infection (3, 4). However, hatched blastocysts were susceptible to PRV infection, which further affected the embryonic development (5). Blastocyst implantation failure is a major cause of pregnancy losses. Intrauterine infection and inflammation are potential causes of implantation failure and miscarriage during early gestation in human (6). Several herpesviruses including cytomegalovirus (7), human simplex virus 1 and 2 (8) are clearly associated with early pregnancy complications *in vivo*. Human herpesvirus-6A infected and altered endometrial cells *in vitro*, which might interfere trophoblast cell attachment, causing failure of embryo implantation (9).

Embryo implantation occurs around day 14 post mating in sows (10). Fetal death is highly dependent on gestation stage, and approximately 30% of embryos are lost during embryo implantation, which is mainly due to abnormal maternal-fetal communication (11). As an important member of herpesviruses family, we have limited knowledge about the direct response of endometrium to PRV exposure at the time of implantation. In the present study, to gain an insight into the pathogenesis of PRV infection during early pregnancy, we established a mouse model of PRV infection through intrauterine inoculation and studied the outcome of embryo implantation.

## 2 Materials and methods

### 2.1 Animals

Female Kunming mice, 8–9 weeks of age, were purchased from Hunan SJA Laboratory Animal Corporation (Changsha, China). Mice were housed under 12 h light and 12 h dark cycles and controlled temperature (23 ± 3°C). Animals had access to feed and water *ad libitum*, and were acclimated for 7 d prior to preparation of pregnancy. All animal procedures were performed with the approval of the Ethical Committee of Animal Experiments, Hunan Agricultural University (approval no. 2021.050).

### 2.2 Intrauterine inoculation of PRV

The PRV-YY strain was propagated in a porcine kidney cell line (PK-15) as described previously (12). Female mice (9–10 weeks of age) were housed with proven-fertile males of the same strain. Vaginal plug was checked in the following day morning, and mice carrying plugs were designated as gestation day 0.5 (GD0.5). The timed-pregnant mice were anesthetized with isoflurane inhalation at GD2, and a small incision was introduced on the back to expose the uterine horn. A volume of 50 μL of PRV with either 10^4^ or 10^5^ TCID_50_ units was injected into each uterine horn using an insulin syringe. Control mice were inoculated with 50 μL of PK-15 cell culture supernatant. After suturing, each animal was placed in an individual cage. Clinical symptoms of mice were monitored three times every day. At GD6, mice were sacrificed, and number of implantation site was recorded. Tissues including thymus, spinal marrow, brain, uterus carrying embryo implantation site, and ovary were collected.

### 2.3 Detection of viral infectivity

To determine the PRV infectivity, viral nucleic acid was detected in the collected tissues from the PRV-inoculated dam and control mice at GD6. Tissues were homogenized in PBS using a homogenizer (FastPrep-24 Instrument, MP Biomedicals, Irvine, CA, United States). Viral DNA was extracted using a DNA Extraction kit (TIANGEN Biotech, Beijing, China) according to the manufacturer’s instructions. Total DNA (500 ng per sample) was subjected to real-time quantitative PCR (RT-qPCR) along with a standard plasmid. The plasmid containing a glycoprotein E (gE) gene, was serially diluted and subjected to RT-qPCR to generate a standard curve using specific primers as described previously (12). The number of PRV copies was determined from the standard curve by converting the corresponding Ct value.

### 2.4 *In situ* viral DNA hybridization

PRV infection was further confirmed in brain and uterus carrying embryo implantation site by *in situ* hybridization. A DNA probe covering 572 bp of the PRV gD genomic portion was constructed using a PCR-digoxigenin (DIG) Probe Synthesis Kit (Roche Diagnostics, Basel, Switzerland) according to the manufacturer’s procedures. The probe was verified by agarose gel electrophoresis. *In situ* hybridization was performed as described previously (13) with modifications. After deparaffinization and rehydration, tissue sections were pretreated with 10 μg/mL proteinase K at 37°C for 15 min and fixed with 4% cold paraformaldehyde (PFA) for 5 min. Slides were rinsed in distilled water and prehybridized with buffer containing 50% (v/v) deionized formamide in 4 × saline-sodium citrate (SSC). Then sections were incubated with hybridization buffer containing 200 ng/mL DCH-DIG probe at 95°C for 6 min to denature DNA, and cooled on ice for 1 min, then incubated at 42°C overnight in a hybrid furnace. The slides were then serially washed with graded SSC solutions, equilibrated in Buffer I (100 mM Tris, 150 mM NaCl, pH 7.5), blocked with 1% blocking reagent (Boehringer-Mannheim, Indianapolis, IN) in Buffer I, and incubated with anti-DIG-alkaline phosphatase antibody (Roche) at room temperature for 1 h. Sections were washed three times in Buffer I and equilibrated in Buffer III (100 mM Tris, 150 mM NaCl, pH 9.5). Color development was performed using NBT/BCIP (Roche) in Buffer II (100 mM Tris, 100 mM NaCl, 50 mM MgCl_2_, pH 9.5) at 4°C overnight. Slides were rinsed in Buffer I, counterstained with a neutral red staining solution and mounted using polyvinylpyrrolidone mounting medium (Beyotime Biotechnology, Haimen, China).

### 2.5 Histology staining

Uterine sections were fixed in 4% PFA for further histological analysis. Tissues were embedded in paraffin, and sectioned. Sections were immersed in xylene to remove paraffin, rehydrated using decreasing grades of ethanol (absolute to 50%) followed by distilled water, and stained with hematoxylin and eosin. Slides were imaged using a microscope. Histology slides were reviewed by a board-certified veterinary pathologist.

### 2.6 RNA extraction, reverse-transcription, and qPCR

Frozen uterine tissues were homogenized in 1 mL TRIzol reagent (Invitrogen, CA, United States). Total RNA was extracted according to the manufacturer’s instructions, and reverse transcribed to cDNA using Vazyme HiScript®III Q RT SuperMix for qPCR (+gDNA wiper) (Vazyme Biotech Co., Ltd., Nanjing, China). RNA levels were determined by using Vazyme ChamQTM SYBR®qPCR Master Mix (Vazyme) on a 7,500 Fast Real-time PCR System (Applied Biosystems, Foster City, CA, United States). Specific primer sequences were shown in Supplementary Table S1. Levels of PCR products were normalized to the housekeeping gene *β*-actin using the 2^−ΔΔCT^ method.

### 2.7 TUNEL assay

TUNEL assay was performed using a DNA Fragmentation Detection Kit (Beyotime) according to the manufacturer’s instructions. Briefly, the deparaffinized sections were re-hydrated in gradient alcohols, and fixed in 4% PFA followed by PBS wash. Slides were incubated with 20 μg/mL proteinase K (Beyotime) at 37°C for 20 min, and rinsed in PBS twice. TUNEL staining was then performed followed by counterstaining with DAPI (BOSTER, Wuhan, China). The images were collected using a fluorescence microscope.

### 2.8 Western blot

Frozen uterine samples were homogenized in RIPA buffer (Solarbio, Beijing, China) with protease inhibitor cocktails (Sigma-Aldrich, Saint Louis, MO, United States) and the total proteins were extracted. Concentration of protein was measured using a Pierce BCA protein assay kit (Thermo Fisher Scientific, Waltham, MA, United States). Equivalent amounts of total protein (20 μg) were separated on SDS-PAGE gels and transferred to polyvinylidene fluoride membranes (Immobilon®-PSQ, Bedford, MA, USA). Membranes were blocked in 5% non-fat milk in PBS with Tween 20 for 2 h at room temperature and incubated with indicated antibodies in blocking buffer at 4°C overnight. Primary antibodies included: rabbit anti-Bax antibody (1:3,000, ab182733, Abcam, Cambridge, MA, United States), rabbit anti-Bcl-2 (1:3,000, ab182858, Abcam), and HRP-conjugated mouse anti-beta Actin (1:10,000, KC-5A08, Kangcheng Biotechnology, Shanghai, China). After rinsing with TBST, HRP-conjugated goat anti-rabbit secondary antibody (7074S, Cell Signaling Technology, MA, United States) was incubated on membranes in 5% milk and bands were developed with ECL reagent (KGP1127; Keygen Biotech, Nanjing, China) using ChemiDoc™ XRS+ system (Bio-Rad Laboratories, Hercules, CA, United States). ImageJ was used for densitometry of Western blots.

### 2.9 Statistical analysis

One-way ANOVA was performed to determine statistically differences followed by Tukey’s multiple comparisons test using GraphPad Prism 6 (GraphPad Software Inc., San Diego, CA, United States). *p* value under 0.05 was considered statistically significant.

## 3 Results

### 3.1 PRV infection in mice via intrauterine inoculation

**Figure 1:**
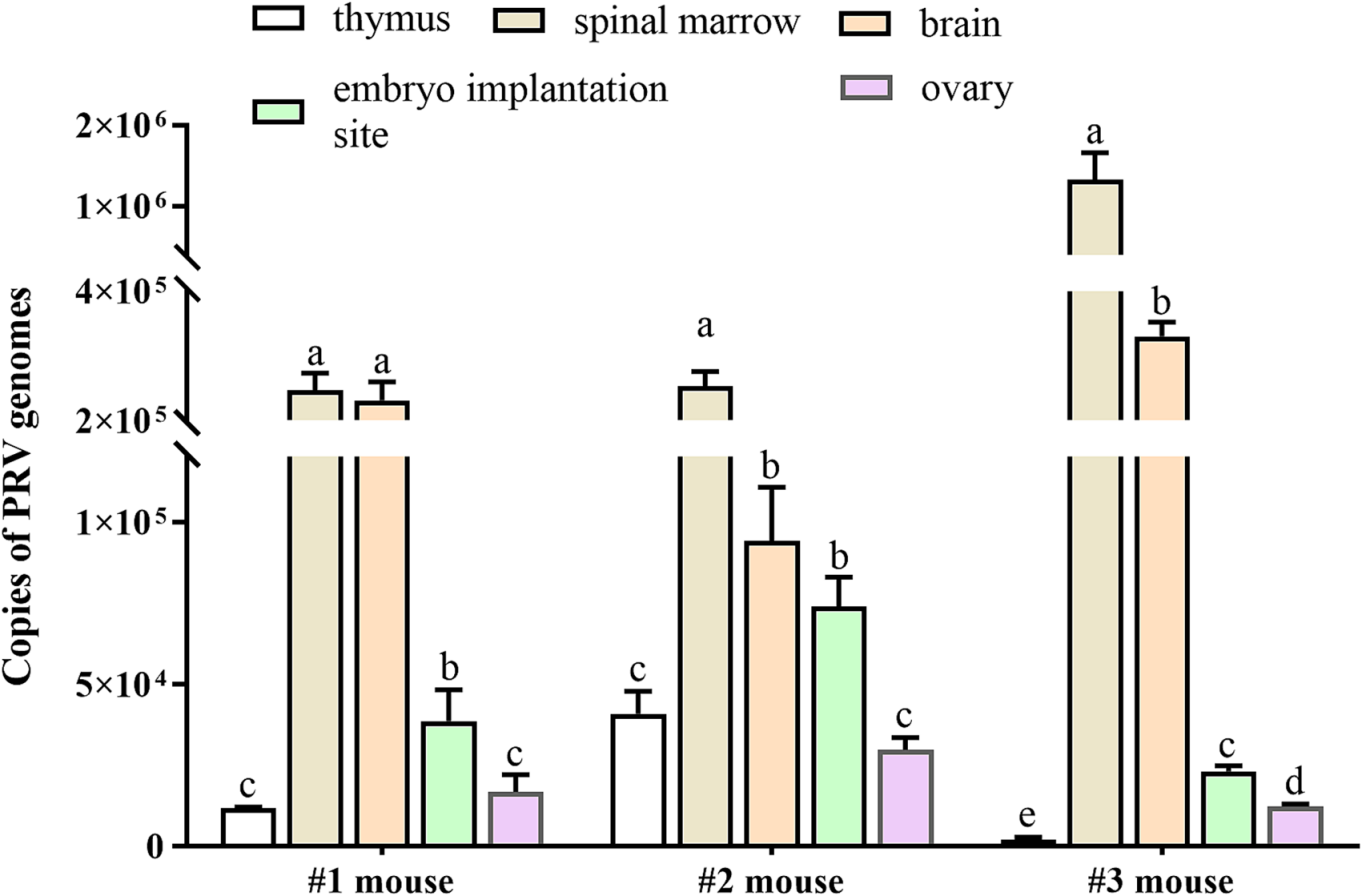
Viral loads of PRV in tissue samples of mice inoculated with PRV. Pregnant mice were inoculated with 50 μL of PRV suspension (a titer of 10^5^ TCID_50_) at GD2, and thymus, spinal marrow, brain, uterus carrying embryo implantation site and ovary were collected at GD6. PRV viral loads were measured in the collected tissues by real-time qPCR in both PRV-inoculated mice (*n* = 3) and Mock-treated group (*n* = 3). Data represent mea*n* ± SD; different lowercase letters indicate significant difference between tissues, and *p* < 0.05.

To investigate whether the PRV would establish infection and cause adverse pregnancy outcomes in pregnant mice, 10^4^ or 10^5^ TCID_50_ of PRV was inoculated in the pre-implantation mice at GD2 via intrauterine route, respectively. The PRV-inoculated dams exhibited clinical signs of pseudorabies around GD6, and death occurred soon after developing clinical symptoms (Supplementary Figure S1A). Viral nucleic acid was detected in different tissues by RT-qPCR. The copy numbers of PRV genomes were measured in the samples collected from the 10^5^ TCID_50_ PRV-inoculated dams (*n* = 3). The results showed that PRV viral loading exhibited discrepancy in different tissues from individual mouse, which was abundant in spinal marrow and brain tissues, and small amounts of PRV genomes were also obtained in thymus, ovary, and uterus carrying embryo implantation site (Figure 1). No viral nucleic acid was detected in tissues from the Mock-treated mice (*n* = 3). The presence of viral DNA in brain was confirmed using *in situ* hybridization, but no positive signal was observed in the embryo implantation site of uterus in the PRV-inoculated pregnant mice (Supplementary Figures S1B–E). These findings indicate that PRV can infect mice via intrauterine inoculation.

### 3.2 PRV infection impaired embryo implantation in mice

**Figure 2:**
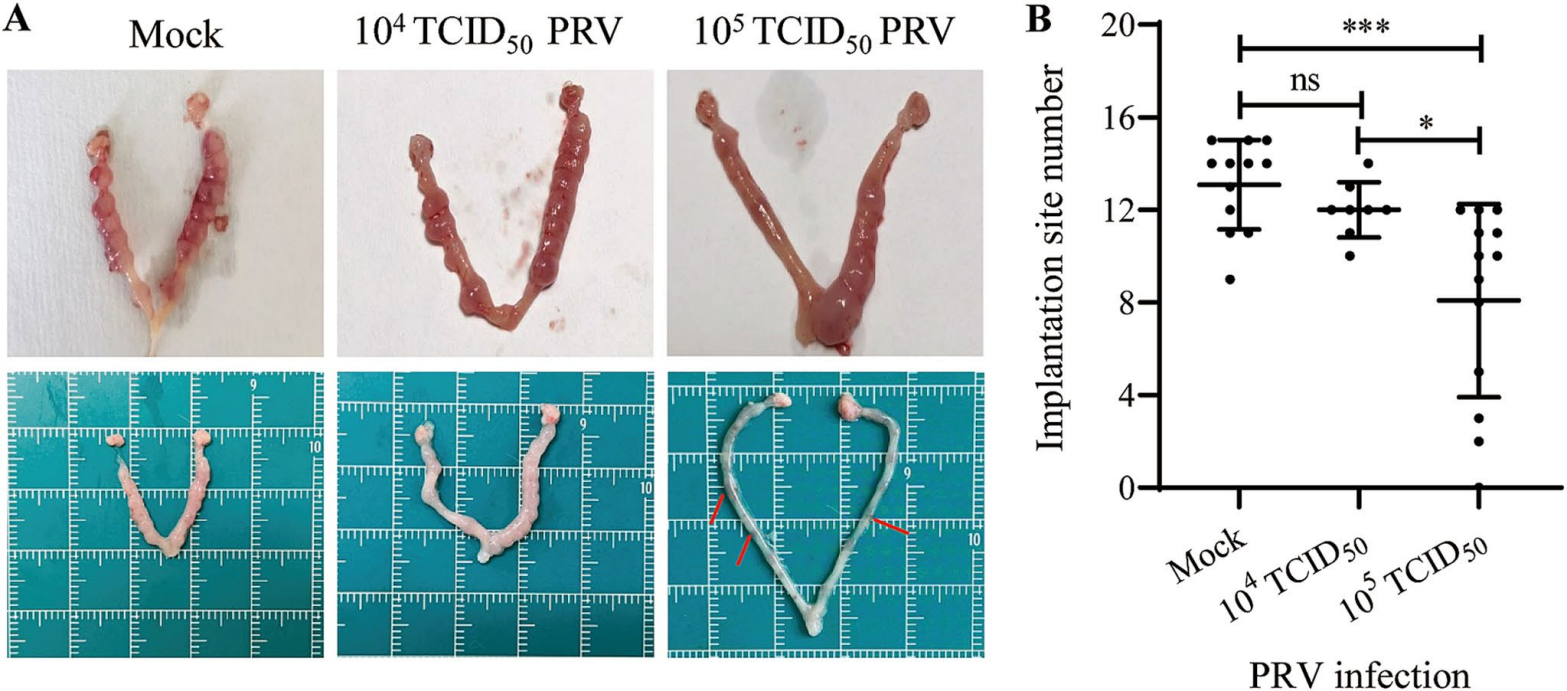
Effect of intrauterine inoculation of PRV on embryo implantation in mice. **(A)** Uterine gross morphology of mice inoculated with Mock, 10^4^ TCID_50_ or 10^5^ TCID_50_ of PRV via intrauterine inoculation; arrows indicate the degenerated implantation sites. **(B)** Analysis of the number of implantation site between groups. Data represent mea*n* ± SD. ‘ns’ means no significance, * *p* < 0.05, and *** *p* < 0.001.

To determine whether PRV infection cause subfertility in mice, the uteri were examined at GD6 (4 days post-inoculation, dpi) to evaluate the ability of embryo implantation. The results showed that inoculation with 10^5^ TCID_50_ of PRV significantly decreased mouse embryo implantation (Figure 2A). The mock-treated mice showed an average 13.08 ± 1.93 of implantation sites per mouse (*n* = 12). Mice infected with 10^4^ TCID_50_ PRV showed an average 12.00 ± 1.20 implantation sites (*n* = 8). However, dams infected with 10^5^ TCID_50_ of PRV carried significantly fewer implantation sites (8.15 ± 4.02, *n* = 13) than the Mock group (*p* < 0.001) (Figure 2B). These results demonstrate that intrauterine inoculation of PRV impairs mouse embryo implantation.

### 3.3 PRV induced inflammation in mouse uterus

**Figure 3:**
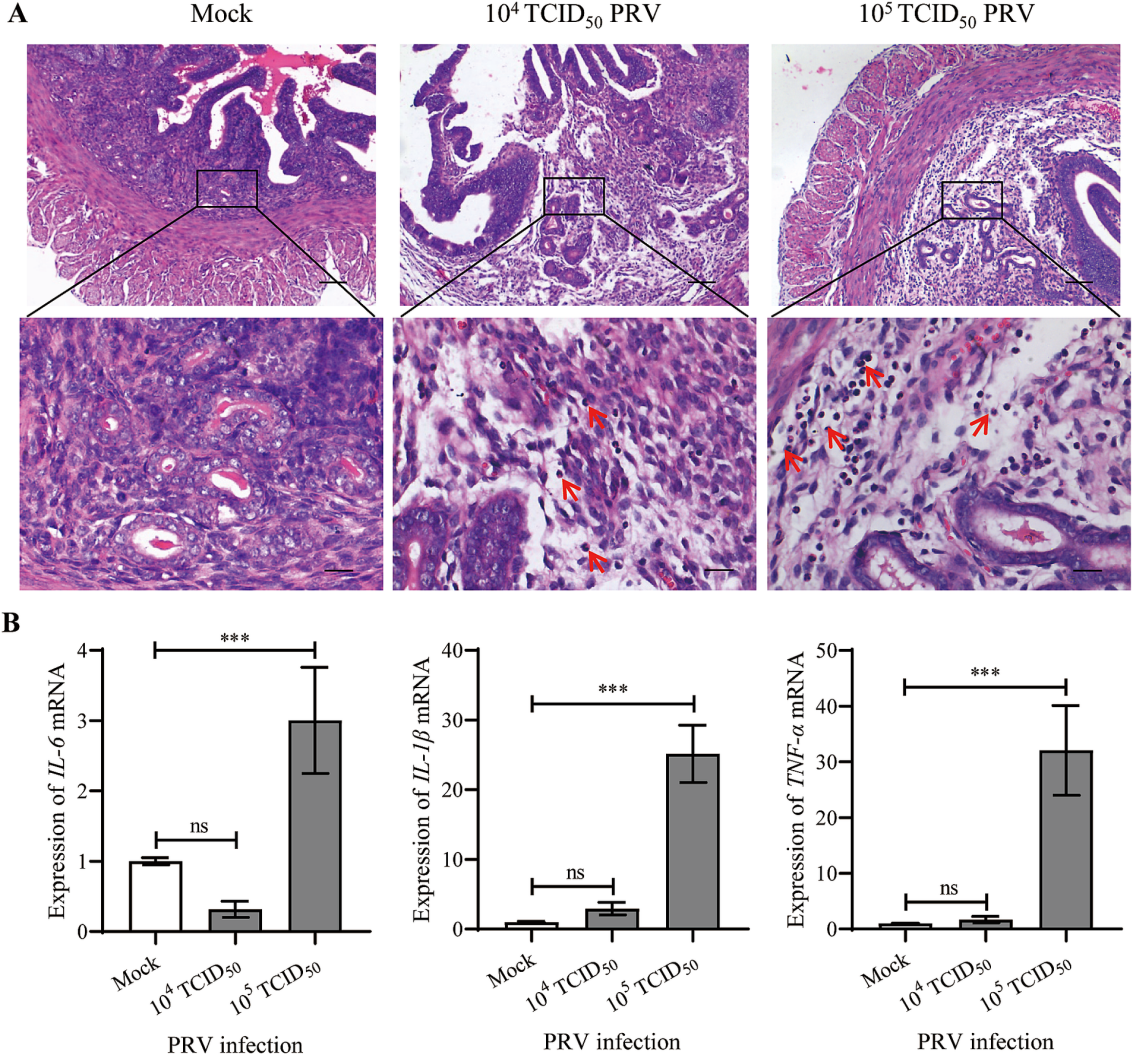
Intrauterine inoculation of PRV induced inflammation in mouse endometrium. **(A)** Representative images of H.E. staining at the implantation sites of Mock and PRV-inoculated mice; arrows indicate inflammatory cells infiltrating in the endometrium; bars for upper and lower panel were 200 μm and 50 μm, respectively. **(B)** Expression of IL-6, IL-1β and TNF-*α* mRNA in uteri. Data represent mea*n* ± SD (*n* = 3). ‘ns’ means no significance, and *** *p* < 0.001.

H.E. staining was performed to evaluate the histopathological changes in uterus. Compared to the Mock group, the stromal decidua of endometrium was relatively looser in the 10^4^ TCID_50_ PRV-infected mice with a few inflammatory cells infiltrating (arrow indicate), which was more severe in the 10^5^ TCID_50_ PRV-infected group (Figure 3A). Nuclei of the majority inflammatory cells were rod-shaped, like neutrophils. We speculated that the decreased ability of embryo implantation was due to the intrauterine inflammatory changes. Therefore, the expression of proinflammatory cytokines including IL-6, IL-1β and TNF-*α* in uterus was further analyzed by qPCR. The results showed that transcription levels of the three inflammatory factors were significantly increased in 10^5^ TCID_50_ PRV-inoculated uterus (*p* < 0.001), and the increase in IL-1β and TNF-α was greater than that of IL-6 (Figure 3B). These results indicate that intrauterine inoculation of PRV induces inflammation and causes endometrial defects in mice.

### 3.4 PRV promoted apoptosis in mouse uterus

**Figure 4:**
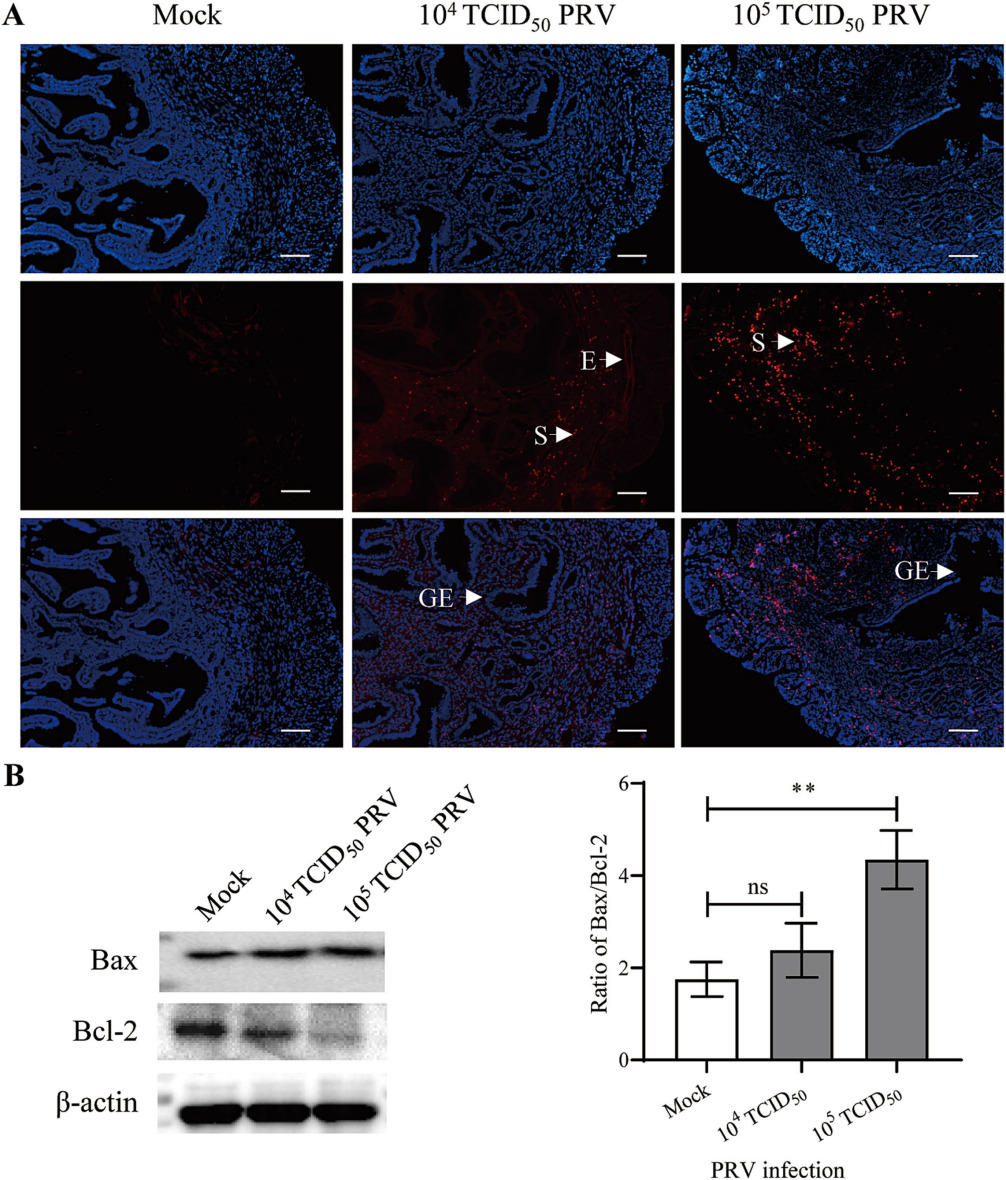
Intrauterine inoculation of PRV promoted apoptosis in mouse endometrium. **(A)** Representative image of TUNEL staining in mouse uterus; Red and blue fluorescent signals represented TUNEL positive staining for apoptotic cells and DAPI nuclear staining, respectively; E: endothelium, S: stroma, GE: glandular epithelium; bar = 200 μm. **(B)** Expression of Bcl-2 and Bax in uterus carrying embryo implantation site, data represent as mea*n* ± SD (*n* = 3). ‘ns’ means no significance, and ** *p* < 0.01.

Apoptosis of the uterine cells was detected by TUNEL staining. In the Mock group, a few apoptotic cells (red fluorescent signal) were found in the stroma, but a medium and large number of apoptotic cells were observed in stroma and endothelium of the endometrium in 10^4^ and 10^5^ TCID_50_ of PRV-infected animals, respectively; no apoptotic signal was observed in the glandular epithelium (Figure 4A). Bax and Bcl-2 are important pro-apoptotic and anti-apoptotic protein. Western blot assay further showed that the ratio of Bax/Bcl-2 was significantly increased in the 10^5^ TCID_50_ of PRV-infected group compared with the Mock group (*p* < 0.01) (Figure 4B).

## 4 Discussion

PRV, as a major pathogen in pig husbandry, can transmit among diverse animal species, causing wide contamination in the environment of pig farms (14). More than 25 cases of PRV infection in humans have been reported in China since 2017, and these patients started with influenza-like symptoms, quickly developed into neurological symptoms, with some even death (15). PRV infection induced a specific and lethal systemic inflammatory response in a footpad inoculation mouse model, developing a severe pruritus in the foot and become dying at 82 h post-inoculation (16). Ren et al. found that mice intramuscularly inoculated with 10^5^ ~ 10^6^ TCID_50_ PRV-GXLB-2013 produced neurological signs and induced inflammatory injuries in different tissues, promoting the expression of several proinflammatory cytokines, including IL-1β, IL-6, TNF-*α*, interferon-*γ* and monocyte chemoattractant protein-1 (17). In the present study, timed pregnant mice in 10^5^ TCID_50_ PRV-YY group developed obvious neurological syndromes at 3 ~ 4 dpi via intrauterine injection and suffered death soon. However, the pregnant mice in 10^4^ TCID_50_ PRV group did not exhibited clinical symptoms, which is not inconsistent with results reported by others, in which 20% of mice died at 4 dpi (17). The difference in animal age, viral strain, duration and route of inoculation may account for the divergence between the studies.

The PRV-infected dams (10^5^ TCID_50_) contained fewer implantation sites in uteri. Mild histopathological changes were observed in endometria of the PRV-infected pregnant mice, with a fewer number of glandular epithelia and looser stromal structures. The results indicate that endometrial defect contributes to the decrease of embryo implantation in PRV-infected mice. It has been shown that a proper inflammatory reaction is beneficial to establish uterine receptivity and successful implantation (18, 19). However, sustained high levels of proinflammatory factors may cause embryo implantation failure (20). We found considerable inflammatory cells infiltrating in the stromal decidua of implantation sites, with high levels of proinflammatory cytokines expressed in uterine tissues after PRV infection. Therefore, PRV infection may trigger cytokine storms which disturbs the intrauterine cytokine homeostasis in the uterus.

Apoptosis is an important cellular defense mechanism, which regulates proliferation and exclusion of cells. At the time of implantation, the endometrium undergoes morphological and physiological changes, such as angiogenesis, apoptosis, and cell proliferation to attain a receptive state. Mild apoptosis was observed at the implantation site in the Mock-treated mice. Host cells were induced to undergo apoptosis during PRV replication (21). The present results confirmed that PRV infection induced massive apoptosis in stroma of the endometrium. Apoptosis was also obvious in the endothelia. Therefore, further research is needed to investigate the effects of PRV infection on function of vascular endothelium and angiogenesis during placentation following embryo implantation.

Collectively, the present findings have disclosed that intrauterine inoculation of PRV at a titer of 10^5^ TCID_50_ can infect mice and impair embryo implantation, which is associated with endometrial defect caused by cytokine release syndrome and apoptosis.

## Data Availability

The original contributions presented in the study are included in the article/Supplementary material, further inquiries can be directed to the corresponding authors.
