# Supplementary material for: Intrauterine inoculation of pseudorabies virus impairs mouse embryo implantation via inducing inflammation and apoptosis in endometrium
Source: Front Vet Sci. 2024 Oct 31;11:1475400. doi: 10.3389/fvets.2024.1475400 (PMC11562746; doi:10.3389/fvets.2024.1475400)
Supplement: Supplementary file 1 [file Table_1.docx]

Supplementary Material

**Supplementary Table 1** Primers used for qPCR in this study

| **Genes** | **Forword primer (5’-3’)** | **Reverse primer (5’-3’)** | **Accession No.** |
| --- | --- | --- | --- |
| *β-actin* | ACAGGGTGGTGGACCTCATG | GGGTCTGGGATGGAAACTGG | XR_002343817.1 |
| *TNF-α* | ATGCTGGGACAGTGACCTGG | CCTTGATGGTGGTGCATGAG | NM_001278601.1 |
| *IL-6* | TTCCATCCAGTTGCCTTCTTG | GAAGGCCGTGGTTGTCACC | NM_001314054.1 |
| *IL-1β* | GCCACCTTTTGACAGTGATG | TGATGTGCTGCTGCGAGATT | NM_008361.4 |
