# Supplementary figures and images for: Intrauterine inoculation of pseudorabies virus impairs mouse embryo implantation via inducing inflammation and apoptosis in endometrium
Source: Front Vet Sci. 2024 Oct 31;11:1475400. doi: 10.3389/fvets.2024.1475400 (PMC11562746; doi:10.3389/fvets.2024.1475400)

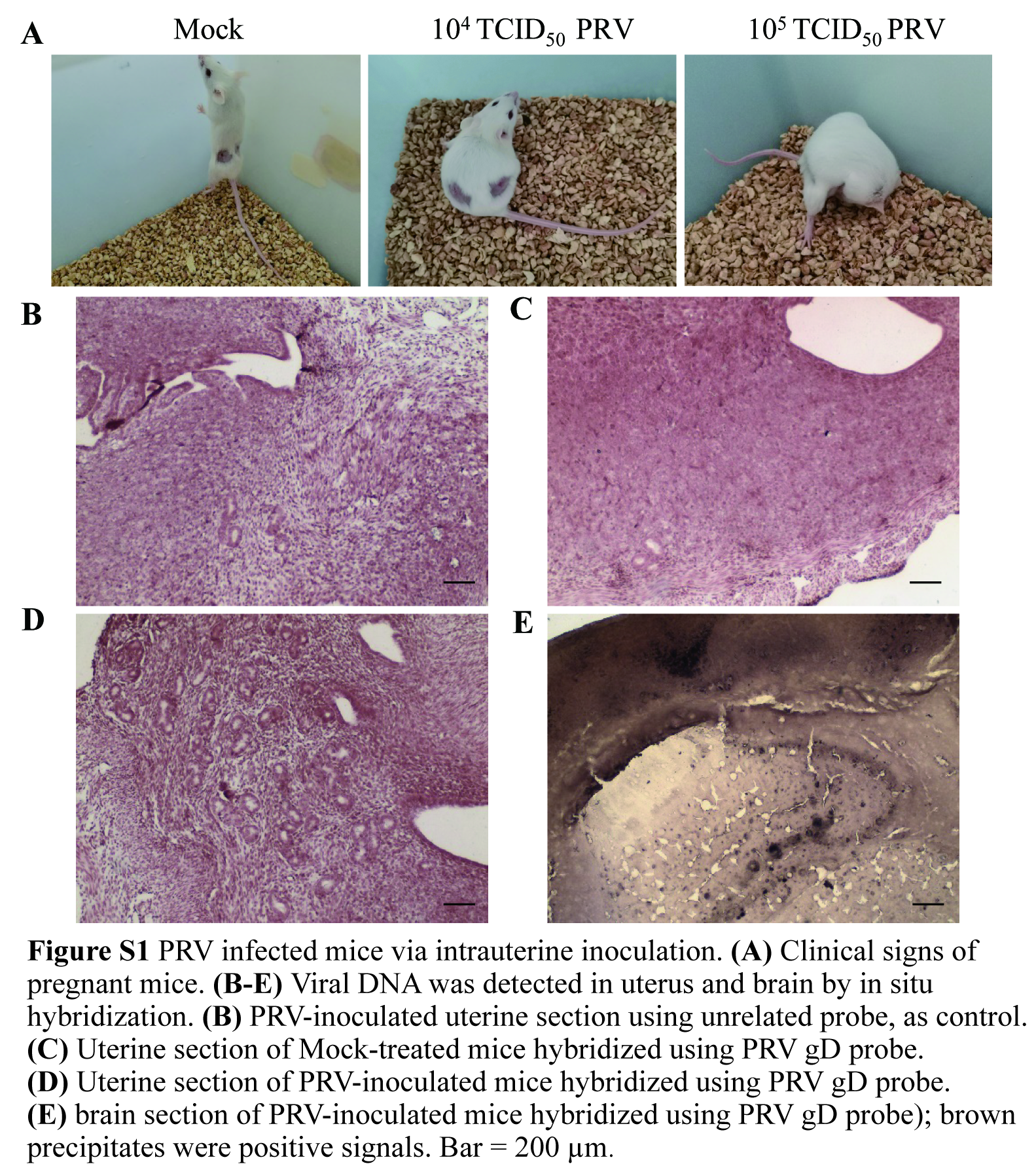

Supplement: Supplementary file 2 [file Image_1.tif]
